# Optimizing hospital-to-home transitions for older persons in rural communities: a participatory, multimethod study protocol

**DOI:** 10.1186/s43058-021-00179-w

**Published:** 2021-07-22

**Authors:** Mary T. Fox, Souraya Sidani, Jeffrey I. Butler, Mark W. Skinner, Marilyn Macdonald, Evelyne Durocher, Kathleen F. Hunter, Adrian Wagg, Lori E. Weeks, Ann MacLeod, Sherry Dahlke

**Affiliations:** 1 School of Nursing, Faculty of Health, York University Centre for Aging Research and Education, York University, HNES suite 343, 4700 Keele St, Toronto, Ontario M3J 1P3 Canada; 2 School of Nursing, Faculty of Community Services, Ryerson University, 350 Victoria Street, Toronto, Ontario M5B 2K3 Canada; 3 School of Nursing, York University Centre for Aging Research and Education, Faculty of Health, York University, HNES suite 343, 4700 Keele Street, Toronto, Ontario M3J 1P3 Canada; 4 Trent School of the Environment, Trent University, 1600 West Bank Drive, Peterborough, Ontario K9L 0G2 Canada; 5 School of Rehabilitation Science, Institute of Applied Health Sciences, Faculty of Health Sciences, McMaster University, Room 428, 1400 Main St. W, Hamilton, Ontario L8S 1C7 Canada; 6 Faculty of Nursing, University of Alberta, 5-293 Edmonton Clinic Health Academy 11405-87 Ave NW, Edmonton, Alberta T6G 1C9 Canada; 7 School of Nursing, Dalhousie University, Room 130, Forrest Bldg., PO Box 15000, 5869 University Avenue, Halifax, Nova Scotia B3H 4R2 Canada; 8 Department of Geriatric Medicine, University of Alberta, 1-198 Clinical Sciences Building, Edmonton, Canada; 9 School of Nursing, Dalhousie University, Room G30, Forrest Bldg. PO Box 15000, Halifax, Nova Scotia B3H 4R2 Canada; 10 Trent/Fleming School of Nursing, Trent University, 1600 West Bank Drive, Peterborough, Ontario K9L 0G2 Canada; 11 Faculty of Nursing, University of Alberta, Edmonton Clinic Health Academy 11405-87 Ave NW, Edmonton, Alberta T6G 1C9 Canada

**Keywords:** Transitional care, Physical functioning, Cognitive functioning, Dementia and caregiving, Intervention adaptation, Rural communities

## Abstract

**Background:**

Transitional care involves time-limited interventions focusing on the continuity of care from hospital to home, to optimize patient functioning and management. Providing interventions, as part of transitional care, that optimize the functioning of older people with dementia is critical due to the small window of opportunity in which they can return to their baseline levels of functioning. Yet prior research on transitional care has not included interventions focused on functioning and did not target older people with dementia in rural communities, limiting the applicability of transitional care to this population. Accordingly, the goal of this study is to align hospital-to-home transitional care with the function-related needs of older people with dementia and their family-caregivers in rural communities.

**Methods:**

In this multimethod study, two phases of activities are planned in rural Ontario and Nova Scotia. *In phase I*, a purposive sample of 15–20 people with dementia and 15–20 family-caregivers in each province will rate the acceptability of six evidence-based interventions and participate in semi-structured interviews to explore the interventions’ acceptability and, where relevant, how to improve their acceptability. Acceptable interventions will be further examined in *phase II*, in which a purposive sample of healthcare providers, stratified by employment location (hospital vs. homecare) and role (clinician vs. decision-maker), will (1) rate the acceptability of the interventions and (2) participate in semi-structured focus group discussions on the facilitators and barriers to delivering the interventions, and suggestions to enable their incorporation into rural transitional care. Two to three focus groups per stratum (8–10 healthcare providers per focus group) will be held for a total of 8–12 focus groups per province. Data analysis will involve qualitative content analysis of interview and focus group discussions and descriptive statistics of intervention acceptability ratings.

**Discussion:**

Findings will (1) include a set of acceptable interventions for rural transitional care that promote older patients’ functioning and family-caregivers’ ability to support patients’ functioning, (2) identify resources needed to incorporate the interventions into rural transitional care, and (3) provide high-quality evidence to inform new transitional care practices and policies and guide future research.

Contributions to the literature

- Preserving and optimizing physical and cognitive functioning in people with dementia is essential to successful hospital-to-home transitions. This study builds knowledge on how to achieve this goal.
- This study will result in a set of acceptable evidence-based interventions for transitional care, promoting the physical and cognitive functioning of older people with dementia during the post-discharge period before implementing and evaluating them for rural transitional care.
- Adapting interventions for this new group of consumers and this new context is vital to ensuring the future implementation, uptake, and effectiveness of the interventions.

## Background

The transition from hospital to home is precarious for older people with dementia (PWD). They are 25% more likely than those without dementia to be readmitted to hospital within 30 days of discharge due to their compromised functional abilities and complex care requirements which, if ill-managed, may result in permanent disability [1, 2]. After hospital discharge, older PWD continue to be at high risk for functional decline, with up to 60% losing their independence in performing activities of daily living such as getting out of bed and dressing themselves [3–5]. Those who do not return to their pre-hospital, baseline level of functioning within 30 days of discharge are unlikely to ever do so [2]. Lack of support during this period is associated with adverse events in this population, including functional decline and related complications (e.g., falls), resulting in high rates of costly rehospitalizations and emergency department visits [6, 7]. In our prior work, we found older people to be highly sedentary after hospital discharge, spending most of their time in bed [8]. Others have found 77% of older people to be less physically active in the first week post-discharge than in hospital [9]. Also, some family-caregivers (FCs) believe it is best for their older relatives to rest, and discourage activity and self-care for fear of exacerbating health problems, and may inadvertently reinforce their relatives’ dependency and deconditioning [10–12].

Transitional care (TC) involves time-limited interventions, focusing on the continuity of care from hospital to home, to optimize patient functioning and management [13, 14]. Providing interventions, as part of TC, that optimize the functioning of older PWD is critical due to the small window of opportunity in which they can return to their baseline levels of functioning. Yet several gaps in care and knowledge, validated in our prior pan-Canadian engagement activities with stakeholders (e.g., FCs, healthcare providers), persist [15].

### Poor fit between transitional care and rural communities

Rural communities have a higher percentage of older people and more people at risk for dementia than urban communities [16]. Older age and dementia are risk factors for functional decline both during and after hospitalization [4] and, independent of illness, older rural community dwellers have poorer functional health, higher rates of disability [17], and higher 30-day hospital readmission rates [18] than their urban counterparts. Rural communities additionally face entrenched health service deprivation (reduced health resources and health system infrastructure), as well as geographical impediments to healthcare access (distance, isolation, and widely dispersed populations), leaving many older people dependent on informal family care [19]. These key features have been overlooked in previous trials evaluating TC and, thus, their findings have limited applicability to rural communities [20]. Furthermore, in several trials, TC was provided from hospital admission up to 3 months post-discharge and involved frequent home visits [21–26]. These TC protocols are unlikely to be feasible given the wide geographical dispersion of patients and the limited health human resources in rural communities and their limited human health resources [27].

### Limited evidence on functional care planning beyond hospital discharge

Our prior systematic review and meta-analysis of interventions designed to preserve older people’s functioning during hospitalization established that PWD who receive these interventions have fewer falls and episodes of delirium, less decline in their ability to perform activities of daily living, shorter hospital stays, and lower rates of discharge to nursing homes, compared to those receiving usual care [28]. The trials included in the review, however, did not continue the interventions after hospital discharge [28]. At 1 and 3 months following discharge, the interventions were not associated with improved functioning [29–32] or hospital readmission rates [28] suggesting that continued intervention is needed as part of TC to reduce functional decline in PWD post-discharge.

In a follow-up systematic review of hospital-to-home TC trials, we established that while TC initiated in hospital significantly reduces hospital readmission and readmission length of stay for older people, the findings have limited applicability because the TC trials excluded PWD and did not focus on functioning [33]. Most TC trials have focused on medication reconciliation, patient education on warning signs of deteriorating health, information transmittal to community providers, and coordination of follow-up appointments [14, 34–39]. To date, few TC trials have addressed the functional needs of older PWD [14, 39]. These findings are especially troubling given that older PWD have more than twice the rate of hospital admission of those without dementia [1], are increasingly discharged home “sicker and quicker” [40, 41], are more vulnerable to breakdowns in the continuity of care during the transfer home, and thus have a great need for TC emphasizing functioning [42]. Older PWD report feeling highly vulnerable after hospital discharge and in need of guidance on how to preserve and restore their functioning while recovering at home [42]. Only three TC trials aimed to improve the functioning of older PWD, but they were conducted with urban dwellers in American [24, 43] and British metropolitan hospitals [44]. Two of the trials found no significant effects on functional outcomes [24, 44] raising questions about patient adherence to the interventions [44] and in the one trial that reported significant effects, the majority of eligible participants (76%) chose not to participate [43] limiting generalizability of the findings and raising doubts about the acceptability of the interventions to older PWD. Acceptability is defined as the desirability of an intervention in addressing a health need or problem [45]. Taken together, these trials underscore the importance of better understanding the perspectives of PWD on interventions aimed at promoting their functioning to optimize intervention implementation, uptake, and effectiveness.

### Poor fit between transitional care and family-caregivers’ needs

FCs provide most post-discharge care for older PWD and are thus crucial for TC to be effective [14]. Yet two thirds of FCs report that their needs are not addressed in TC [14, 46]. Current TC is inadequate, resulting in declines in physical and cognitive functioning in PWD [7, 47], who have more chronic conditions requiring more complex care management [48, 49]. Furthermore, during the transition from hospital to home, older PWD experience increased trouble thinking, remembering, concentrating, and making decisions [50] which complicate caregiving [51]. Their functional abilities and care requirements often differ substantially following hospital discharge than before admission [46]. FCs report feeling unprepared to successfully manage care [14] and have stressed their need for practical caregiving strategies to help their older relatives resume functioning [42, 52].

In summary, TC does not adequately address the function-related needs of older PWD and FCs in rural communities, underscoring the importance of collaborating with them and healthcare providers (HCPs) in adapting interventions targeting the physical and cognitive functions of older PWD so that TC better fits this population’s needs and healthcare context. Adapting (i.e., modifying and/or adding) interventions [53] for new consumers and contexts is vital to ensuring their future implementation, uptake, and effectiveness [45, 54–56].

### Study objectives

The objectives of this study are to (1) explore the perceived acceptability of six evidence-based interventions (hereafter, referred to as interventions) to promote physical and cognitive functioning in older PWD and FCs in rural communities, (2) examine healthcare providers’ (HCPs) perceived acceptability of the interventions, and (3) adapt the interventions for transitional care to fit the function-related needs of PWD and FCs and the rural healthcare context.

### Research questions

The research questions are the following:

1. What perspectives do rural-dwelling older PWD and their FCs have on the acceptability of the interventions to address their function-related needs at home in the post-discharge period?
  - Which interventions fit/do not fit their needs and why?
  - Which interventions require adaptation and how?
  - What are FCs’ perspectives on how the interventions may impact them personally?
2. What perspectives do hospital and homecare HCPs have on the interventions (identified as acceptable to PWD and FCs)?
  - Which interventions do HCPs perceive as acceptable?
  - Which require modification and how?
  - What resources are needed to incorporate the interventions into rural TC?

### Interventions to promote physical and cognitive functioning

The six interventions proposed for TC are derived from prior reviews of trials done in urban acute and post-acute care settings; the interventions target the physical and cognitive functions of PWD that are commonly adversely affected by acute illness and hospitalization [20, 28, 56, 57]. The interventions are evidence-based and have been found to prevent functional decline and related complications in older hospitalized PWD, with statistically significant, small to moderate effects [20, 28, 43, 56]; they thus represent the most promising interventions for TC. They offer practical guidance on how to promote physical and cognitive functioning (while addressing affect) at home after discharge and, because they have been used in other populations with dementia [28, 43, 57], are expected to be applicable to PWD in this study. PWD and FCs are supported in learning about the interventions by HCPs in hospital and they are provided follow-up educational support over the post-discharge period by HCPs at home, promoting use of the interventions and their effectiveness. Because hospital-to-home TC in rural communities marks a particularly pronounced shift from HCP-driven to self- and family-managed care, our study focuses on interventions that PWD and FCs can use on their own at home.

*Physical functioning interventions* specifically target the following:

(i) *Orthostatic tolerance*. Strategies to promote upright physical activity tolerance following a period of inactivity (e.g., continue to progressively stand/walk three times a day; perform low intensity antipostural exercises twice daily; limit recumbent or semi-recumbent position to < 20.5/24 h; balance rest with physical activity; involve PWD in setting physical activity goals tailored to preferences, routines, and personal interests; provide encouragement and support) [20, 56–58]
(ii) *Ambulation*. Strategies to promote safe ambulation (e.g., supervise walking; ensure non-skid footwear is worn; remove trip hazards; if fears falling, walk together) [20, 57]
(iii) *Activities of daily living (ADL)*. Approaches to promote independence in ADL (e.g., encourage PWD to do as much self-care as possible; use verbal and/or visual cues to guide self-care; praise independent task performance; allow PWD time to complete task; if PWD become frustrated, validate feelings, stress the activities that they can do, and move on to a simpler activity; provide rest periods between activities; consider playing music to increase motivation) [20, 43, 57, 59]

*Cognitive functioning interventions* specifically target the following:

(i) *Sleep*. Strategies to promote sleep (e.g., establish consistent bedtime routine; limit time in bed during the day; use behavioral techniques, such as a back massage to assist with physical and mental relaxation; avoid emotional or mental stimulation before bed that may cause anxiety or the inability to relax; if worried, make a plan to deal with it the next day) [20, 60–63]
(ii) *Sensory*. Strategies to promote orientation (e.g., ensure PWD have glasses and hearing aids on; put clocks and calendars within view; ensure proper lighting; minimize environmental distractions and noise to prevent overstimulation and agitation) [20, 43]
(iii) *Communication*. Strategies to promote comprehension and memory (e.g., speak in calm short sentences; ask simple yes/no questions; give PWD time to answer; if having difficulty remembering, use pictures or cues; if anxious about forgetting important information, write reminders down and leave within view of PWD) [43, 64, 65]

## Methods/design

This 3-year multimethod descriptive study is guided by the *Intervention Acceptability* and *Collaborative Intervention Planning Frameworks* as well as an integrated-knowledge translation approach, which bring together intervention adaptation and the principles of community-based participatory research, and prioritize engaging both healthcare consumers and providers when adapting interventions [45, 53]. The frameworks provide direction on how to systematically adapt interventions to align them with consumers’ needs in particular contexts, while maintaining the interventions’ “active ingredients” or “essential elements” (i.e., indispensable components required to improve outcomes), to enhance future implementation, uptake, and effectiveness [45, 53, 66]. Our approach concurs with the Medical Research Council’s emphasis on involving consumers in intervention design; the Council acknowledges that intervention evaluation is often challenged by problems related to acceptability that limit intervention uptake by consumers and intervention effectiveness [55].

Accordingly, two sequential phases of research activities, designed in collaboration with knowledge-users (i.e., PWD, FC, and HCP representatives), are planned to begin in spring 2021. Phase I addresses the first research question. TC *consumers* (older PWD and FCs) will be involved in first adapting the interventions to ensure they meet their function-related needs. Phase II addresses the second research question. TC *providers* (HCPs) will inform modifications to aspects of the interventions, such as mode of delivery (e.g., home visit), to enable their incorporation into rural TC. This sequence allows interventions that are acceptable to consumers to be brought forward to HCPs who can then consider how to deliver them in rural communities. Both phases will be conducted in Southwestern and Northeastern Ontario as well as Eastern and Western Nova Scotia, Canada, where team knowledge-users are located. Ontario and Nova Scotia have substantial rural populations; 1,806,036 Ontarians (14% of the population) and 400,389 Nova Scotians (43% of the population) reside in rural communities [67]. Both provinces have populations older than the national average [67].

### Phase I: Perspectives of older people with dementia and their family-caregivers

#### Sample and setting

The target population consists of older PWD and their FCs. The purposive sample includes English-speaking PWD and FCs who will be identified either in collaborating hospital or homecare centers. Eligible PWDs reside in rural communities (i.e., small towns outside the commuting zones of larger urban centers), in areas accessible by car in < 3 h; are aged 65+ with a diagnosis of any type of dementia; have the cognitive ability to respond to questions; were discharged from hospital to home following admission through the emergency department (thus at higher risk for functional decline) [68] for an acute medical illness or acute exacerbation of a chronic medical condition (people with medical conditions have the highest hospital readmission rates in rural communities) [18]; had a length of stay > 48 h (functional decline begins within 48 h of hospital admission) [69]; and have a FC. PWD will be excluded if they were admitted for palliation or stroke, are unable to walk on discharge, or are prescribed home rehabilitation, which would preclude their consideration of the interventions. PWD transferred to a rehabilitation, long-term, or alternate level of care prior to returning home will also be excluded. Eligible FCs are unpaid relatives, partners, or friends [14] aged 18+, who live with, and are the primary providers of care at home of PWD enrolled in this study.

Between 15 and 20 PWD and 15 and 20 FC interviews will be conducted per province for a total of 30–40 PWD and 30–40 FC interviews, depending on how quickly informational saturation is achieved [70]. This sample size is adequate for the quantitative descriptive purposes of phase I [71] and falls within the range recommended for sufficient and feasible qualitative data collection and management [72–74].

#### Variables and measures

##### Screening measures

The cognitive ability of PWD to respond to questions will be established by a score > 13 on the Mini Mental State Exam (MMSE) based on evidence indicating that people with these scores have been found to respond consistently and accurately to semi-structured interview questions and to be highly receptive to sharing their views with researchers [75]. The MMSE has been found to be a reliable and valid test of cognition [75, 76].

The inability of PWD to walk on discharge will be measured by the corresponding item of the Duke Activity Status Exam (DASI) that inquires about walking indoors [77]. A *no* response indicates inability to perform the activity. The DASI is an established self-report measure of functional capacity that has shown construct validity (manifested by significant correlations with conceptually related scales [78] and physiological measures [77]), as well as internal consistency reliability (Cronbach alpha = .86) [78].

PWD residence in a rural community is determined by the size of the town’s population (< 10,000) and its location outside the commuting zones of larger urban centers (i.e., populations > 100,000) [17]. Other PWD screening measures (e.g., diagnosis of dementia, admitted for a medical illness) will be measured using a standard form. Apart from the MMSE, all PWD screening measures will be completed by proxy FCs to reduce response burden in PWD. Screening measures for FCs (e.g., age 18+) will be measured by self-report using a standard form.

##### Perceived intervention acceptability

PWD and FCs’ perceived intervention acceptability will be measured by a single self-report item (derived from the Intervention Acceptability Scale) that assesses overall acceptability [79]. To reduce response burden, we applied a response tree to the item while maintaining the original response options [80]. The response tree involves first asking respondents to indicate if the intervention would be helpful to them; a *yes no* (rated 0) response is used. Respondents who answer *yes* will then be asked to indicate how helpful the intervention would be to them by selecting one of four response options ranging from *a little bit* (1) to *extremely* (4).

##### Demographic and health characteristics

To describe the sample, PWD demographic (e.g., marital status, region of residence) and health characteristics (e.g., reason for admission) will be collected from proxy FCs (to reduce response burden on PWD) with standard items we have used previously [81, 82] as well as the DASI [77]. FCs will respond to similar standard questions to describe their own demographic characteristics.

#### Data collection procedures

The PI, with assistance from knowledge-users (e.g., administrators) from the collaborating sites, will explain the study via videoconference to hospital discharge planners and home care coordinators and provide them with a summary sheet of the study that includes eligibility criteria. Hospital discharge planners and homecare coordinators will identify potentially eligible participants by pre-screening for study eligibility criteria that are known to the staff and available in the medical record (e.g., diagnosis of dementia) using a standard form. They will introduce the study (using a standard script) and ask participants for permission to give their names to the research nurse (who will be hired locally). The research nurse will contact interested participants to determine eligibility and explain the study and the procedure for obtaining assent/consent.

Using established assent and consent procedures, PWD will be asked to describe what is required of them to participate in the study and any risks, and those able to answer these questions will be asked to sign a consent form [83]. Those unable to answer these questions will be asked if they would like to participate in the study. A “yes” response will be considered assent [84] and the research nurse will contact the legal guardian (if different from the FC) to seek proxy consent. After obtaining assent/consent, the research nurse will confirm eligibility by administering the screening measures/questions (as described above) and arrange the interview for consenting eligible participants. Our prior research, with an enrollment rate of 84%, supports the effectiveness of this recruitment strategy (unpublished data).

Consenting, eligible PWD and their FCs will be interviewed by videoconference (due to current COVID-19 pandemic restrictions). Once the restrictions are lifted, PWD and FCs will be offered the option of having a face-to-face interview. All interviews will be conducted within 30 days after discharge by a research associate (RA) experienced in interviewing this population. PWD will be interviewed first to reduce fatigue, followed by FCs. PWD will be offered the option to have their FCs present (to enhance comfort and promote recall) [85–87]. Interviews will be conducted using a semi-structured interview guide that was developed based on the Intervention Acceptability and Collaborative Intervention Planning Frameworks, and includes questions derived from their constructs (e.g., perceived appropriateness, burden, self-efficacy in performing the behaviors required in using the interventions). We have previously used similar guides and accompanying interview materials successfully [82, 88]. These will be pilot tested for clarity, comprehension, and time commitment with 3–5 eligible PWD and FCs. Using the guide, the RA will engage PWD in discussing the interventions in relation to their needs in resuming physical and cognitive functioning at home during the post-discharge period, and FCs in relation to their needs in supporting these functions. The RA will also ask what other needs PWD and FCs have and suggested strategies beyond the six interventions that may address them. FCs will additionally be asked to comment on how the interventions may impact them personally.

The interviews will be conducted in line with best interviewing practices for PWD, such as conducting interviews early in the day at times convenient for PWD and FCs, using gender neutral visual illustrations (e.g., someone walking) to describe and remind participants of the topic under discussion, using verbal cues to focus attention (e.g., “look at this picture with me”), and providing preludes to challenging questions to alleviate anxiety about answering them (e.g., “some people might find the next question a little hard to answer”) [86]. The RA will describe each intervention, administer the overall intervention acceptability item, and solicit participants’ perspectives on why it is/is not acceptable in addressing their needs and which aspect(s) of the interventions should be adapted and how. The descriptions, designed specifically for older PWD, include simple, step-by-step written explanations displayed in large font, in simple lay terms for PWD and FCs to follow. To minimize burden, interviews have been designed not to exceed 30 min for PWD and 60 min for FCs [89]. Interviews will be audio-recorded and transcribed.

#### Data analysis

Descriptive statistics, in accordance with each variable’s level of measurement, will be used to analyze demographics and responses to the overall acceptability item. Conventional qualitative content analysis of the interview transcripts will be conducted concurrently with data collection (facilitated by NVivo 12) and will focus on identifying interventions perceived by PWD and FCs as acceptable, as well as any needs and strategies not accounted for in the six interventions [90, 91]. Interventions will be considered acceptable if they have a mean rating > 2 (i.e., midpoint of the response scale) and participant narratives reveal that, even if adaptations are necessary, the interventions meet PWD and FCs’ needs [92]. For interventions rated unacceptable (rated < 2), participants will be asked for suggestions on improving their acceptability and fit with their needs. Content analysis will entail creating preliminary codes and, through an inductive iterative process, synthesizing them into meaningful, hierarchically organized categories and sub-categories [91]. For each code, category, and subcategory, definitions will be created and the relationships among them delineated; illustrative quotes for each will be documented. Data will be examined for patterns in participants’ narratives (e.g., by region, reason for hospital admission, gender, functional capacity, whether home care is received) and acceptability ratings using conceptual matrices [91] and memos [93]. Strategies to ensure trustworthiness will be employed (e.g., documenting an audit trail will enhance confirmability, independent data analysis by researchers and the involvement of knowledge-users in the interpretation of findings will enhance credibility, detailed methodological description will enhance dependability; and the inclusion of participant demographic data such as region and province of residence will enhance transferability [94, 95]).

In collaboration with knowledge-users, a list of additional needs and strategies will be created. The literature on these strategies will be reviewed; if there is evidence that they support functioning, we will develop corresponding intervention logic models and add them to the set of interventions for phase II. Synthesized from empirical literature, intervention logic models describe an intervention’s goals (i.e., what it aims to achieve), activities (i.e., what it entails), mode of delivery (e.g., home visit), dose (e.g., how often), benefits (e.g., improved outcomes), and the human and material resources needed to provide it. For interventions considered by PWD and FCs as acceptable but requiring modifications, the recommended modifications will be integrated into the intervention logic models and brought forward to phase II [53, 96]. Overall, phase I findings will highlight interventions that are acceptable to PWD and FCs, fit their needs and, hence, are likely to be used.

### Phase II: Perspectives of healthcare providers

#### Sample and setting

The purposive, criterion-based sample of HCPs will be stratified (4 strata) by role (clinician vs. decision-maker) and employment location (hospital vs. homecare), in each province. The stratification is done because TC bridges hospital and home settings, is delivered by clinicians (e.g., nurses, social workers), and is administrated by decision-makers (e.g., managers, directors). Eligible HCPs work in either Southwestern or Northeastern Ontario or Eastern or Western Nova Scotia and serve PWD living in these rural communities. Casual, temporary, part-time (working < 21 h/week), or agency HCPs are ineligible [97].

Two to three focus groups per stratum, with 8 to 10 HCPs per group, will be held in each province (for a total of 8–12 focus groups per province) until informational saturation is achieved, based on the rationale that a minimum of 2 focus groups per stratum is needed to achieve saturation [98]. Separate focus groups will be held with HCPs within each of these 4 strata (clinician vs. decision-maker, hospital vs. homecare) to maintain within-group homogeneity. This number of focus groups falls within the range recommended for sufficient and feasible qualitative data collection and management [99]. Between 32–60 clinicians and 32–60 decision-makers will be recruited in each province for a total of 128–240 healthcare providers. Recruitment strategies will be initiated by HCPs who are members of the research team and include presenting the study at staff meetings and through email, and posting flyers at the sites and on their social media accounts. Strategies to promote participation, such as holding focus groups outside of work time and providing a modest honorarium, will be employed.

#### Variables and measures

##### Screening measures

Screening variables for healthcare providers (e.g., works in Ontario or Nova Scotia) will be collected using a standard self-report form.

##### Perceived intervention acceptability

HCPs’ perceived intervention acceptability will be measured by the Intervention Acceptability Scale. The self-report scale has a set of items assessing HCPs’ perspectives on the interventions’ appropriateness, effectiveness, risks, and ease of use [79, 100]. A five-point scale ranging from *not at all* (0) to *very much* (4) is used in the rating and the total score is obtained by taking the mean of the item responses. The Intervention Acceptability Scale provides a systematic approach to assess HCPs’ perceptions of an intervention that can then be explored in greater depth in group discussion [79, 101]. The scale has demonstrated internal consistency reliability (alpha > .80) and factorial validity [79, 101].

##### Demographic and professional characteristics

HCP demographic (e.g., age) and professional (e.g., role, employment location) characteristics will be measured using a questionnaire with standard questions previously used by our team in order to describe the sample [101].

### Data collection

Consenting HCPs will be invited to focus groups to discuss the acceptability of, and suggest modifications to, the interventions brought forward from phase I to enable their incorporation into rural TC. Prior to the focus groups, we will provide participants with a package containing the intervention logic models and Intervention Acceptability Scales, and the questionnaire on demographic and professional data; depending on preference, HCPs may choose to complete them online or in hard copy. To control for possible order effects in responses to the scale, the sequence of the Intervention Acceptability Scales (and corresponding intervention logic models) will be randomized using a computer-generated randomization scheme, resulting in the generation of different orders of the scales. In collaboration with knowledge-users, we will review the scores (average rating, range of values) of each intervention’s acceptability and finalize the semi-structured interview guide for the focus group.

Focus groups lasting up to 2 h will then be scheduled. Due to COVID-19 restrictions, the focus groups will be held via videoconference. Once the restrictions are lifted, HCPs will be offered the option of having face-to-face focus groups. At the focus groups, the RA will summarize the phase I findings and HPCs’ ratings of the interventions to contextualize the focus group discussion. The RA will engage HCPs in a semi-structured discussion on the acceptability of the interventions and their fit with the rural healthcare provider context. Questions are guided by the Intervention Acceptability and Collaborative Intervention Planning Frameworks and, together with accompanying interview materials will be pilot tested for clarity, comprehension, and time commitment in one focus group. The questions will prompt HCPs to discuss: (1) why inventions were rated unacceptable (mean rating score < 2) [92]; (2) facilitators and barriers to delivering them in rural communities; (3) the human and material resources required to deliver them; and (4) suggestions for modifying them to enable their delivery. Strategies will be used to ensure equal engagement by all team members (e.g., turn-taking to ensure all voices are heard). Responses will inform modifications to aspects of the interventions (e.g., mode of delivery) to enable their delivery by HCPs. We have successfully used this approach previously in adapting interventions [92, 101].

### Data analysis

Descriptive statistics, in accordance with each variable’s level of measurement, will be used to analyze demographics and the perceived acceptability of the interventions to HCPs (ratings will be available for a total of 128–240 participants: 8–10 participants/focus group × 4 strata × 2–3 focus groups/stratum × 2 provinces). Transcripts of the focus group discussions will be content analyzed [90, 91] using the qualitative criteria for trustworthiness described in phase I. HCPs’ perspectives will be summarized within each stratum and compared across strata (clinician vs. decision-maker, hospital vs. homecare) to identify any patterns of similarity and difference. This context-sensitive approach will illuminate if and how employment location, professional roles, gender, and region shape perceptions of the acceptability of the interventions, and barriers and facilitators in their future implementation. Findings will enable the identification of: commonalities in perceptions across regions that are likely to be applicable elsewhere, interventions viewed unfavorably and intervention aspects requiring modification, and resources and strategies enabling HCPs to deliver the interventions as part of rural TC. A comprehensive list of barriers, facilitators, recommended modifications, and resources will be created in collaboration with knowledge-users on our team (PWD, FC, and HCP representatives). Suggested modifications will be reviewed to ensure they do not drift significantly from the essential elements of the interventions or PWD and FCs’ recommended adaptations. The intervention logic models will be revised to reflect changes in intervention aspects and recommend resources to enable the delivery of the interventions as part of rural TC [53, 96].

### Knowledge translation

This project employs an integrated-Knowledge Translation (*i-KT)* approach, complemented by a comprehensive *end-of-grant KT plan*. Our *i-KT objective* is to maximize collaboration among researchers and knowledge-users in all aspects of the research to enhance the relevance of the findings, and to ensure that knowledge-users are invested in using and disseminating the findings. *To achieve our i-KT objective, our approach* employs a collaborative model of shared governance that emphasizes egalitarian dialog and actively engages knowledge-users throughout the research process (e.g., knowledge-users contributed to the development of the project objectives, research questions, and design). Over the course of the project, we will continue to draw on their insights to enhance our KT plan. To maximize the project’s relevance to knowledge-users, they will be represented on HCP and PWD-FC advisory committees for reviewing progress, advising on the conduct of the proposed activities, interpreting the findings, and devising knowledge mobilization strategies. We will additionally work jointly to develop an *i-KT evaluation plan* that will help foster facilitators to collaboration and address potential barriers [102]. To mitigate power differentials (e.g., between different health professions, between consumers and providers), meetings will be organized so that all members may have their voices heard and constructively participate. Depending on PWD and FC communication preferences (e.g., one-to-one vs group meetings), a combination of teleconferences, videoconferences and email will be utilized. PWD team members will be invited to identify how they wish to be involved and to guide the team by sharing their personal knowledge of living with dementia.

Our *end-of-grant KT objectives* include (1) disseminating findings on the perceived acceptability of the interventions, (2) raising awareness on the resources needed to successfully incorporate the interventions into rural TC, and (3) informing future research evaluating the effectiveness of the interventions for TC in rural Canada as well as future research adapting other interventions for non-traditional research populations. To meet these objectives, *dissemination strategies* will involve communicating key findings to relevant audiences through plain language summary and policy briefs of key findings, tailored to end-users [103], disseminating intervention logic models describing the interventions, and reporting on the systematic collaborative process we used for adapting the interventions for rural TC. These will be introduced to targeted knowledge-users at interactive web-conference dialogs for policy-makers, PWD, FCs, and HCPs, in addition to presentations at scholarly conferences for researchers. Webinars will be posted on the York University Centre for Aging Research & Education (YU-CARE) website. Findings will be published in high-impact open-access journals to make them available to national and international audiences conducting research in, or delivering TC to rural communities. The intervention logic models can be used to train clinicians in implementing the interventions in research, evaluating their fidelity of implementation, and to provide guidance to researchers on how to implement the study’s systematic process for adapting interventions. A press release of the findings will be organized through the communications department at York University.

## Discussion

Functional decline is one of the most disabling and life-altering risk factors associated with older people’s acute hospitalization and recovery [104] and PWD in rural communities are particularly affected [16]. The preservation and optimization of physical and cognitive functioning in PWD and the seamless transition of care are recognized by patients, FCs, and HCPs as essential in the successful management of dementia and other chronic diseases [48]. This study will build knowledge on how to achieve these goals.

Our collaborative approach to intervention adaptation engages PWD in designing their own care, thereby maximizing the acceptability of the interventions to improve functioning during the highly vulnerable post-discharge period. Applying this systematic adaptation process will result in a set of acceptable interventions for TC, promoting the physical and cognitive functioning of older PWD during the post-discharge period before implementing and evaluating them for rural TC. Specifically, assessing the acceptability of the interventions prior to incorporating them into TC will highlight intervention aspects requiring modification to enhance their future implementation, uptake, and effectiveness [45, 105]. The study will provide high-quality evidence for researchers, HCPs, and policy-makers to inform new, innovative TC research, practices, and policies for older PWD and FCs in rural communities. We will use this evidence to guide our future research testing the effectiveness of the acceptable interventions.

We anticipate that, overall, the interventions will be perceived as meeting the function-related needs of PWD and FCs but that modifications in the doses and modes of delivering the interventions by HCPs will be required (e.g., providing them via phone for people who live too far from a home care center to qualify for its in-home services). The project will generate intervention logic models to support implementation of the interventions with fidelity in future rural TC research and practice. The use of multiple sites will enhance transferability of the findings to similar settings nationwide. The findings will (1) increase the capacity of PWD and FCs to use the interventions (e.g., interventions that address PWD and FCs’ function-related needs are more likely to be used), (2) increase the capacity of HCPs to implement the interventions (e.g., interventions aligned with the rural practice context are more likely to be provided), and (3) inform TC policy (e.g., briefs will package findings on the resources required to integrate the interventions into rural TC).

## Data Availability

N/A.

## Abbreviations

COVID-19: Coronavirus disease 2019
FC: Family-caregiver
HCP: Healthcare provider
PWD: People with dementia
RA: Research associate
TC: Transitional care
